# CRISPRa-Mediated Increase of OPA1 Expression in Dominant Optic Atrophy

**DOI:** 10.3390/ijms26136364

**Published:** 2025-07-02

**Authors:** Giada Becchi, Michael Whitehead, Joshua P. Harvey, Paul E. Sladen, Mohammed Dushti, J. Paul Chapple, Patrick Yu-Wai-Man, Michael E. Cheetham

**Affiliations:** 1 UCL Institute of Ophthalmology, Faculty of Brain Sciences, London EC1V 9EL, UKharveyjoshua90@gmail.com (J.P.H.); psladen25@googlemail.com (P.E.S.); 2 William Harvey Research Institute, Faculty of Medicine and Dentistry, Queen Mary University of London, London EC1M 6BQ, UK; m.dushti@qmul.ac.uk (M.D.); j.p.chapple@qmul.ac.uk (J.P.C.); 3 Pharmacology & Toxicology Department, Faculty of Medicine, Kuwait University, Kuwait City P.O. Box 24923, Kuwait; 4 John van Geest Centre for Brain Repair, University of Cambridge, Cambridge CB2 0PY, UK; py237@cam.ac.uk; 5 MRC Mitochondrial Biology Unit, Department of Clinical Neurosciences, University of Cambridge, Cambridge CB2 0XY, UK; 6 Cambridge Eye Unit, Addenbrooke’s Hospital, Cambridge University Hospital NHS Foundation Trust, Cambridge CB2 0QQ, UK; 7 Moorfields Eye Hospital NHS Foundation Trust, London EC1V 2PD, UK

**Keywords:** optic atrophy, CRISPR, retinal ganglion cell, mitochondria, CRISPR activation, gene expression, OPA1, mitochondrial fusion, gene editing, alternative splicing

## Abstract

Dominant Optic Atrophy (DOA) is the most common inherited optic neuropathy and presents as gradual visual loss caused by the loss of retinal ganglion cells (RGCs). Over 60% of DOA cases are caused by pathogenic variants in the *OPA1* gene, which encodes a mitochondrial GTPase essential in mitochondrial fusion. Currently, there are no treatments for DOA. Here, we tested the therapeutic potential of an approach to DOA using CRISPR activation (CRISPRa). Homology directed repair was used to introduce a common *OPA1* pathogenic variant (c.2708_2711TTAGdel) into HEK293T cells as an in vitro model of DOA. Heterozygous c.2708_2711TTAGdel cells had reduced levels of *OPA1* mRNA transcript, OPA1 protein, and mitochondrial network alterations. The effect of inactivated Cas9 fused to an activator (dCas9–VPR) was tested with a range of guide RNAs (gRNA) targeted to the promotor region of *OPA1*. gRNA3 and dCas9–VPR increased OPA1 expression at the RNA and protein level towards control levels. Importantly, the correct ratio of *OPA1* isoform transcripts was maintained by CRISPRa. CRISPRa-treated cells showed an improvement in mitochondrial networks compared to untreated cells, indicating partial rescue of a disease-associated phenotype. Collectively, these data support the potential application of CRISPRa as a therapeutic intervention in DOA.

## 1. Introduction

Dominant Optic Atrophy (DOA) is the most common inherited optic neuropathy, and it is mainly characterised by the progressive loss of retinal ganglion cells (RGCs). The prevalence of DOA varies between populations, with 1 in 25,000 people affected in north England and 1 in 12,000 people affected in Denmark due to a founder effect [1,2,3]. The disease progresses to exhibit characteristic phenotypes, including bilateral and symmetrical optic disc pallor, colour vision deficits, decreased visual acuity, and cecocentral scotoma [4,5,6]. Over 60% of DOA cases are caused by pathogenic variants in the *OPA1* gene [7,8,9,10]. The human *OPA1* gene encodes for a ubiquitously expressed dynamin-related GTPase with a mitochondrial targeting sequence that directs the protein to the inter-membrane space and cristae [11,12,13]. Pathogenic variants are spread throughout the gene length, with a cluster of missense variants affecting the GTPase or dynamin central domains [14,15]. The most commonly reported pathogenic *OPA1* variant is c.2708_2711del, which has been reported in Germany, France, Belgium, the UK, and Denmark [2,13,16]. This variant leads to a frameshift resulting in an early termination codon three amino acids downstream of the deletion site, p.R905*, leading to loss of protein expression and a haploinsufficiency phenotype.

Located on chromosome 3, *OPA1* is a large gene, spanning more than 90 kb, and it is composed of 30 exons, with 3 of these, exons 4, 4b, and 5b, being alternatively spliced to produce 8 isoforms of the protein OPA1 that have tissue-specific patterns of expression [11,17]. The retina, for example, shows high expression of isoform 1, 2, and 7 [18]. Additionally, OPA1 is also proteolytically cleaved by either OMA1 at the S1-site or YME1L at the S2-site [19,20,21]. This allows for the eight transcript isoforms to be able to produce both a full-length OPA1 protein (l-form) and a short one (s-form), with l-forms containing exon 4b being fully processed into s-forms [20].

In mammalians cells, the different *OPA1* isoforms are required, both alone and in combination, for the execution of all OPA1 functions [22]. OPA1 is essential in the fusion of the inner mitochondrial membrane; ablation of the protein has shown around 50% of reduction in fusion, while OPA1 overexpression caused mitochondrial tubulation and elongation [23,24]. Both l- and s-forms of OPA1 complement each other in the rescue of mitochondrial fusion activity, demonstrating the importance of maintaining not only the expression of the correct isoform ratios but also the balance of their proteolytic cleavage [20]. Additionally, OPA1 is responsible for the maintenance of mtDNA, with OPA1 proteins containing the peptide encoded by exon 4b, found to be directly involved in this function [25]. This peptide of OPA1 was reported to maintain the mitochondria transcription factor A and bind with mtDNA D-loop, leading to increased expression of mtDNA-encoded respiratory proteins and, ultimately, the recovery of mitochondrial bioenergetics in *OPA1* null cells [26]. The oligomerisation of OPA1 is involved in narrowing cristae junctions and promoting ATP synthase dimerization, which is necessary to control cristae biogenesis and morphology [27,28]. As the cristae is a main site of oxidative phosphorylation, OPA1 has also been associated with efficient OXPHOS function. Interactions were found between OPA1 and complexes I, II, and III, suggesting the protein to be potentially regulating OXPHOS function by stabilising these complexes [29,30]. In OPA1-deficient cells, the addition of any single isoform was found to restore cristae structure, demonstrating this function to be less variant-specific [22].

Currently, DOA has no approved treatments. In recent years, gene augmentation has been developed as a therapeutic approach to DOA, with OPA1 supplementation showing a protective effect on RGCs [18,31]. However, the increased expression of a single isoform would not be able to rescue the full range of OPA1 functions, which are achieved by the correct balance of all the isoforms in both their l- and s-forms. It is challenging to successfully deliver multiple isoforms using a restricted vehicle, such as an AAV capsid. CRISPR activation (CRISPRa) is a CRISPR-based system whereby Cas9 endonuclease is inactivated and fused to a transcriptional activator. Here, we test the hypothesis that CRISPRa could be used as a potential approach to treat OPA1 haploinsufficiency. By targeting the CRISPRa system upstream of the gene transcription start site (TSS) with suitable guide RNAs (gRNA), the activator could increase the expression of all OPA1 isoforms, thus restoring full protein function. A gene-edited cell line containing the c.2708_2711del variant was created and used to test a range of gRNAs for their ability to increase OPA1 expression. The phenotypic rescue effect of the CRISPRa system was also investigated through the analysis of mitochondrial network organisation.

## 2. Results

### 2.1. DOA HEK293T Cell Model Has Decreased OPA1 RNA and Protein Levels

**Figure 1:**
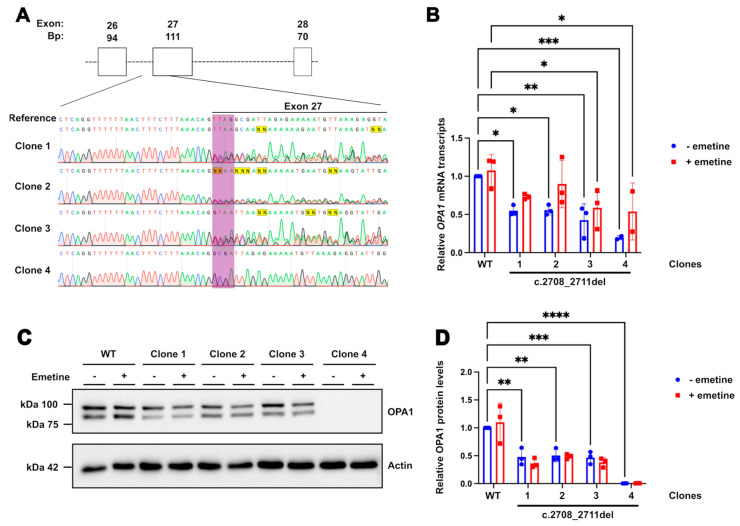
**Characterisation of c.2708_2711del HEK293 cell line.** (**A**) Sanger sequencing results from the heterozygous and homozygous clones obtained after CRISPR edit, with reference WT sequence above. The bases targeted for deletion are highlighted in purple. Exon 27 is overlined in black. (**B**) Quantification of *OPA1* transcript levels in edited cells compared to WT through qPCR. Bars indicate means ± SD. A total of 50 µg/mL of emetine was added to (+) cell samples for 4 h prior to RNA extraction. *GAPDH* and *Actin* were used as reference genes. *p* values were determined through two-way ANOVA. Values between untreated (blue bars) and emetine-treated samples (red bars) showed no significant difference in two-way ANOVA. (**C**) Western blots showing OPA1 protein, both l- and s-forms (upper and lower bands, respectively). (**D**) Quantification of total OPA1 levels (sum of OPA1 l- and s-forms) shows a relative decrease in edited cells. Actin stain used as a loading control. A total of 50 µg/mL of emetine was added to (+) cell samples for 4 h prior to cell lysis. A total of 8 µg of protein was loaded for each sample. * *p* ≤ 0.05; ** *p* ≤ 0.01; *** *p* ≤ 0.001; **** *p* ≤ 0.0001.

In order to test the CRISPRa system in an isogenic easily expandable and transfectable cell line, HEK293T cells were CRISPR gene-edited to knock-in the common *OPA1* c.2708_2711del pathogenic variant [13]. Control (WT) HEK293T cells were nucleofected with a gRNA targeting a PAM site overlapping the intended change in combination with a HDR template used to direct the correct base pair deletions (Figure 1A; Supplementary Figure S1). The template was designed to complement the forward strand of the DNA while including the TTAG deletion, with asymmetric homology arms at both ends to improve efficiency. Individual single cell-derived colonies were isolated and screened through Sanger sequencing of a PCR amplicon encompassing the intronic regions upstream and downstream of exon 27. Clones 1–3 were identified with the correct heterozygous deletions and selected for RNA and protein analysis (Figure 1B). *OPA1* mRNA levels in these cells were shown to be decreased by approximately 50% in the edited cells compared to WT, a predicted outcome as the TTAG deletion at exon 27 has been reported to produce a truncated mRNA transcript that is degraded by nonsense mediated decay (NMD) (Figure 1B) [32]. Emetine, an NMD inhibitor, was added to parallel samples for 4 h to assess the effect of NMD on *OPA1* mRNA. The addition of emetine increased the levels of *OPA1* transcripts compared to the untreated samples; however, this change did not reach statistical significance (Supplementary Table S1), indicating the likely involvement of NMD in the degradation of the mutant transcript despite the limited sample size and treatment time. OPA1 protein levels, quantified by the combined measurement of the two immunoreactive bands on Western blots that represent the s- and l-forms of the protein, were also reduced by approximately 50% in heterozygous cells compared to WT, as expected by the degradation of the truncated protein, and no immunoreactivity corresponding to OPA1 truncated fragments was detected (Figure 1C,D). The treatment with emetine did not lead to an increase in OPA1 protein level in any of the samples, indicating that any protein produced by the c.2708_2711del mRNA is likely unstable and rapidly degraded. Given the similar results seen in all three heterozygous clones, Clone 1 was selected for further work to model DOA.

In addition, the CRISPR edit generated another clone, Clone 4, which produced a clean sequence trace missing the area corresponding to the TTAG, indicating a homozygous deletion. *OPA1* mRNA levels in this line were approximately 20% of WT levels, and no protein was detected, indicating complete degradation of the truncated protein produced by both alleles (Figure 1B–D). The protein was not rescued by the addition of emetine, demonstrating the clone to have a completely null OPA1 background.

### 2.2. OPA1 Haploinsufficiency Leads to Changes in Mitochondrial Network Organisation

**Figure 2:**
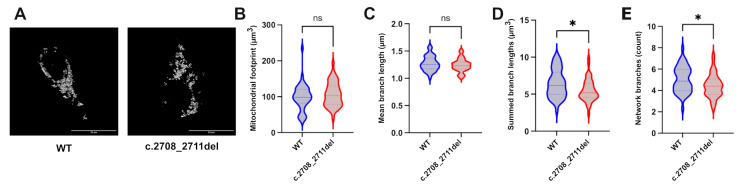
**Mitochondrial network analysis of WT and c.2708_2711TTAGdel cells.** (**A**) Representative images of the maximal intensity of WT and c.2708_2711del HEK293T cells stained with TOMM20. Scale bar represents 20 µm. Analysis was achieved using the Fiji MiNa plugin, which provided measures of (**B**) mitochondrial footprint; (**C**) mean branch lengths; (**D**) summed branch lengths; and (**E**) network branches. *p* values were determined through unpaired t test with Welch’s correction. A total of 45 cells were analysed per sample. * *p* ≤ 0.05; ns = not significant.

The effect of reduced OPA1 function on mitochondrial fusion has been reported in several models [33,34]. Patient-derived monocytes with the c.2708_2711del variant were reported to have an abnormal distribution of mitochondria compared to the homogenous distribution in control samples [8]. Similarly, HeLa cells transfected with a siRNA targeting the GTPase region of OPA1 were reported to have progressive fragmentation of mitochondrial networks [33]. To test for this phenotypic hallmark of loss of OPA1 function, the c.2708_2711del cell line was stained for the mitochondrial marker TOMM20 (Figure 2A), and the resulting mitochondrial networks were analysed using the ImageJ MiNa plug-in (Figure 2). The output produced a 3D-skeleton map of the mitochondria in the cell, allowing for analysis of the distance between each mitochondrion and their relative space in the cell. The summed branch lengths and the number of branches in the mitochondrial network were decreased in c.2708_2711del cells compared to control cells (Figure 2D,E). In the context of the 3D-skeleton map of the mitochondria, this indicates a reduction in mitochondrial connectivity and a decrease in the total distance between them in the cell, suggesting a fragmentation of the mitochondrial network. The mitochondrial footprints and mean branch lengths were not significantly different between the heterozygous knock-in and isogenic control cells (Figure 2B,C).

### 2.3. CRISPRa Improves OPA1 RNA and Protein Levels

**Figure 3:**
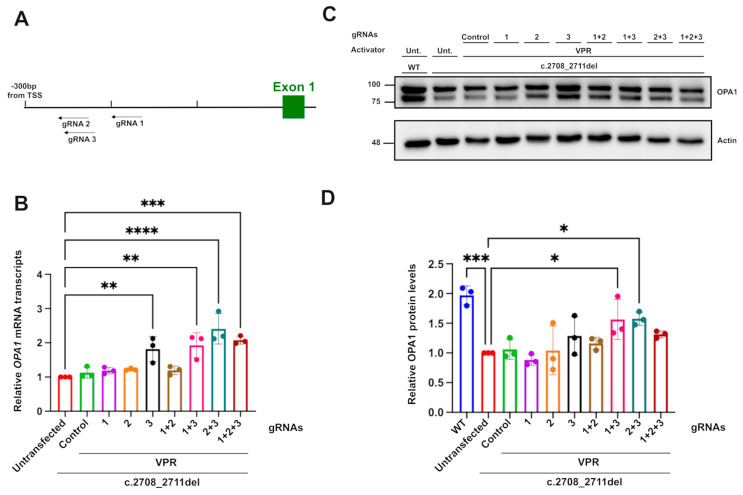
***OPA1* transcript and protein upregulation mediated by dCas9–VPR.** (**A**) Diagrammatic representation of the position of gRNAs in relation to OPA1’s transcription start site. (**B**) Relative *OPA1* mRNA transcript upregulation measured through qPCR, with *ACTIN* and *GAPDH* used as reference genes. (**C**) OPA1 protein expression detected through Western blot and (**D**) quantification of the relative OPA1 protein level using actin immunoreactivity as a reference protein. A total of 8 µg of protein was loaded for each sample. *n* = 3. *p* values were determined through one-way ANOVA. * *p* ≤ 0.05; ** *p* ≤ 0.01; *** *p* ≤ 0.001; **** *p* ≤ 0.0001.

To develop the CRISPRa system in HEK293T cells, different gRNAs were tested in combination with a dSpCas9 fused to a VPR activator [35]. gRNAs were designed using CHOPCHOP (https://chopchop.cbu.uib.no/), with design settings establishing an approximate targeting range of 300 to 150 bp upstream of the TSS. This was established following the comparison of CRISPRa libraries and published reports, which provide a broader range of 0 to around 1 kb upstream of the TSS [35,36,37,38,39,40]. The range was reduced in this design, as the large computational analysis performed in the generation of the CRISPRa library by Horlbeck et al. [39] showed a decrease in gRNA efficiency past 400 bps upstream of the TSS. Furthermore, the same analysis showed an increased trend in CRISPRa activity for guides between around 100 and 300 bp upstream of the TSS. Additional analyses were also performed on the predicted CRISPRa activity with different factors, such as gRNA length, with a slight trend showing increased activity, with longer gRNAs going up to 23 bp [39]. Therefore, the parameters were set to guides of 22 bp. The top three candidate guides with those criteria were selected for use, with two of these, guides 2 and 3, overlapping by 19 bp (Figure 3A). The surrounding histone composition of the area surrounding the position of the gRNAs chosen was also examined on UCSC. The guides were found to be in an area with a peak of H3K27ac in both retinal and HeLa cell tracks, indicating an open chromatin configuration, possibly due to an active enhancer region (Supplementary Figure S2), which has been shown to increase the relative activation efficiency of the CRISPRa machinery [41]. The VPR activator was selected for its robust efficiency in a wide range of different cell models, and it has been reported to produce stronger upregulation compared to other activators, such as VP64 [35,42]. Stronger activators, such as SAM, were considered to be potentially too strong, given their main use in overexpression systems and the potential deleterious effects of OPA1 overexpression.

The transfection of dCas9–VPR with gRNA3 only or in combination with gRNA1 induced around a two-fold increase in OPA1 transcripts relative to untransfected and guide-less transfected c.2708_2711TTAGdel cells. This increase produced transcript levels comparable to WT cells. Combinations of gRNA 2 with gRNA 3 were also found to significantly increase the relative transcript levels despite the guides’ almost total overlap, except for three bases. The effect of the guides on the protein level, assessed through Western blotting, confirmed the upregulation effect, with the combination of guide 1 and 3 producing an approximately 1.5-fold increase in OPA1. The increases in OPA1 protein were less pronounced compared to transcript levels; this was potentially due to the restricted treatment time of the cells with the CRISPRa system, limiting the manifestaton of the full post-transcriptional effect. Together, these results show that CRISPRa can increase OPA1 expression close to physiological levels seen in isogenic control unedited cells, thereby suggesting that it might be able to rescue DOA-associated phenotypes.

### 2.4. CRISPRa’s Effect on OPA1 Isoform Ratios

**Figure 4:**
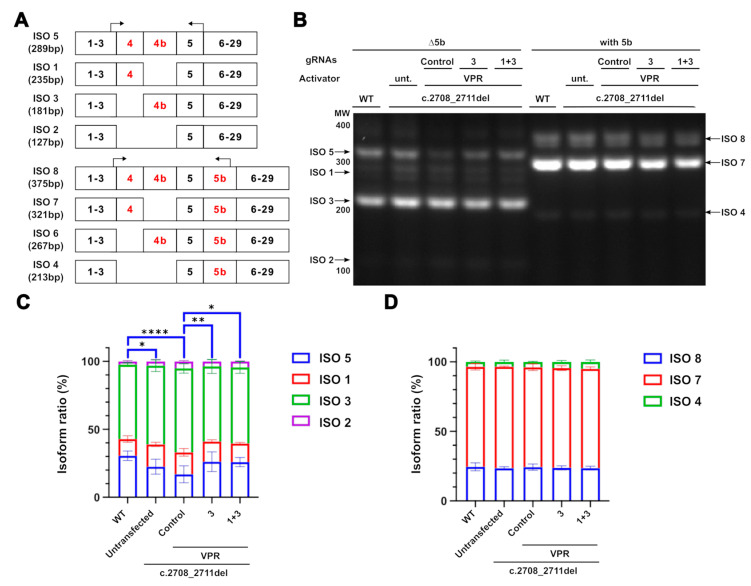
**Maintenance of OPA1 isoform ratios in CRISPRa-treated cells.** (**A**) Diagrammatic representation of OPA1 isoforms with alternatively spliced exons marked in red. Two sets of RT-PCR primers were used to separate isoforms containing and excluding exon 5b, with these primers marked by the arrows. The product size for each primer was marked accordingly. (**B**) RT-PCR isoform products separated on a 2% agarose gel. Black arrows indicate the bands detected (**C**,**D**) Each band was quantified as a percentage of the total transcripts for each amplicon. *n* = 3. Only isoform 5 showed any significant differences between the conditions and is marked in blue. *p* values were determined through two-way ANOVA. * *p* ≤ 0.05; ** *p* ≤ 0.01; **** *p* ≤ 0.0001.

Upregulation of OPA1 expression by CRISPRa offers the potential advantage of maintaining the homeostatic expression of OPA1 isoforms, given the system targets upstream of alternative splicing, at the transcription level. The upregulation of OPA1 should allow the cell to regulate splicing. To investigate how CRISPRa might affect isoforms produced through alternative splicing, the isoform ratios of both the control and the CRISPRa-treated samples were compared. Isoform 6 was undetectable by RT-PCR and could therefore not be quantified. All other isoforms were found to be expressed at a similar ratio across all conditions, except for isoform 5 (Figure 4B–D). The c.2708_2711del line had significantly lower expression of this isoform compared to WT cells (Figure 4C). This indicates that within the reduced OPA1 transcripts present in the c.2708_2711del cells compared to the WT cells, the ratio of isoform 5 is reduced further compared to the other isoforms. In contrast, the CRISPRa system containing gRNA3 alone, or with 1 and 3 together, increased the level of isoform 5 expression. This demonstrates that the system is not only capable of increasing OPA1 expression; as CRISPRa acts upstream of the alternative splicing, the cell inherently regulates the splicing to restore homeostatic ratios.

### 2.5. CRISPRa Rescues Cellular Phenotyes Associated with the Loss of OPA1 Function

**Figure 5:**
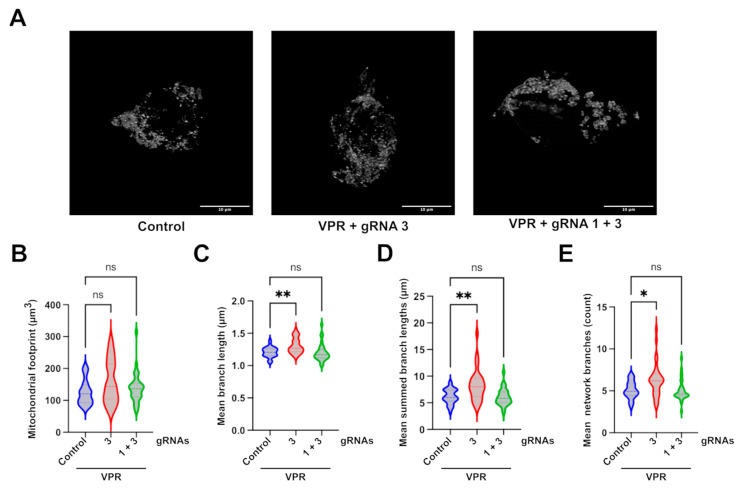
**Mitochondrial network analysis of CRISPRa-treated cells**. (**A**) Representative images of the maximal intensity of c.2708_2711del HEK293T cells treated with different CRISPRa systems and stained with anti-TOMM20. Mitochondrial network analysis of transfected cells identified through intrinsic GFP fluorescence from the dCas9–VPR plasmid and mCherry fluorescence from the gRNA plasmids, achieved through 3D z-stack analysed by the Fiji MiNA plugin. Scale bar represents 10 µm. The analysis produced values for (**B**) mitochondrial footprint; (**C**) mean branch lengths; (**D**) summed branch lengths; and (**E**) network branches. *p* values were determined using the Kruskal–Wallis test. A total of 30 cells were analysed per sample from one experiment. * *p* ≤ 0.05; ** *p* ≤ 0.01; ns = not significant.

Having established a change in mitochondrial networks in the engineered HEK293T c.2708–2711del cell line, TOMM20 immunoreactivity (Figure 5A) was used to assess the effect of CRISPRa on mitochondrial networks. The analysis of transfected cells, identified through GFP and mCherry fluorescence, revealed that dCas9–VPR and gRNA3 significantly increased almost all of the mitochondrial network parameters assessed, except for the mitochondrial footprint (Figure 5B–E). The means of summed branch lengths and network branches, were both significantly increased, indicating an improvement in measures previously reduced in the edited line compared to control cells. This improvement was also observed for the mean branch lengths, which was not significantly decreased in the cell line characterisation compared to isogenic WT cells but suggests a general shift of improved mitochondrial network formation after transfection with dCas9–VPR and guide 3.

## 3. Discussion

In this study we demonstrate the potential of CRISPRa as a therapeutic approach to DOA. To develop this approach and establish its efficacy as a treatment, we CRISPR-engineered a HEK293T cell line containing the most common *OPA1* pathogenic variant in DOA, c.2708_2711del. The heterozygous deletion of 4 bps at exon 27 causes haploinsufficiency of OPA1, which was confirmed at the RNA and protein level in the edited cells. This haploinsufficiency was corrected at the mRNA transcript level and improved at the protein level with the use of dCas9–VPR. Increased activity of CRISPRa using multiple guides has been reported in the literature, both through large-scale analysis in CRISPRa libraries as well as in numerous CRISPRa studies [39,43]. When comparing single and multiple gRNAs in this model, the combination of guides 1 and 3 increased OPA1 expression at the mRNA and protein levels. Interestingly, the combination of gRNA 2 and 3 also increased the levels of *OPA1* transcript more than gRNA 3 alone, despite their target site overlapping. gRNA2 had no effect on OPA1 upregulation on its own, indicating that gRNA3 is likely the main gRNA causing the increase.

The analysis of *OPA1* isoforms in the c.2708_2711del and WT lines demonstrated that isoforms 1 and 7 were expressed at similar levels in both cell lines. These are the most abundant in retinal tissue, and their overexpression has been reported to rescue some of the DOA-associated phenotypes [18]. Expression of isoform 5 was observed to be significantly decreased in the DOA model line, suggesting that the reduction of *OPA1* expression could influence the pattern of splicing. The relative reduction in isoform 5 was rescued using the CRISPRa system, which acts upstream of the splicing machinery, has potential for the cells to naturally restore the homeostatic pattern of *OPA1* isoform expression. It is worth noting that while isoform 5 has not been widely studied in the context of DOA, it has been reported as the most abundant OPA1 isoform in the brain, indicating that the reduction of its expression could have an effect on the mitochondria in the optic nerve [17]. As these experiments were conducted on HEK293T cells, the specific pattern of isoform expression is not as directly relevant to the expression found in RGCs; however, the rescue provided by CRISPRa suggests a potential additional benefit of this therapeutic strategy for the disease phenotype.

The level of OPA1 needed for phenotypic rescue in OPA1 haploinsufficiency has not yet been established; therefore, it was hypothesised that a partial recovery of OPA1 levels might be sufficient to rescue DOA-associated phenotypes. To establish this, disease-associated hallmarks were first investigated in the engineered cell line and assessed for change after CRISPRa. Fragmented mitochondrial networks are a widely reported phenotype observed in many DOA cell models [19,20,33,44]. The transfection of an siRNA targeting OPA1’s GTPase domain in a HeLa cell, for instance, showed progressive punctuated mitochondrial network fragmentation compared to the filamentous network shown in scramble controls [33]. This was corroborated by a similar analysis performed in OPA1-null mouse embryonic fibroblasts [20]. This phenotype was also observed in our engineered line, with shorter summed branch lengths and less network branches, indicating a more fragmented network. The application of CRISPRa using gRNA3 was successful in increasing the parameters affected in the comparison between WT and c.2708_2711del cells, along with increasing the general mean branch lengths, indicating an overall rescue of network fragmentation. In contrast, the combination of gRNA1 and 3 did not provide any significant rescue in mitochondrial network parameters. Although there was a shift in the distribution of the data collected compared to the untreated cells, the overall effect of this combination of guides was too variable and the effect size too small to statistically show a difference from the untreated group. It is not clear why this combination of guides that can successfully increase OPA1 transcript and protein and restore isoform balance was less effective at improving mitochondrial networks than the single guide, which led to similar improvements in OPA1 protein and isoforms. It might reflect the difficulty of imaging mitochondrial networks in transfected cells or variability between individual cells at the level of CRISPRa-mediated OPA1 upregulation and rescue.

Collectively, the data support the possible application of CRISPRa as a potential therapy for DOA. The successful upregulation of *OPA1* at the RNA and protein levels in a haploinsufficient cell line demonstrates that the OPA1 genomic region is accessible for the CRISPRa system to bind and interact with the promotor. The rescue of a DOA-associated phenotype, whilst restoring similar patterns of OPA1 isoform expression to WT cells, further supports the advantages of this approach, which could rescue most, if not all, *OPA1* functions in patients with *OPA1* haploinsufficiency. Further studies on patient-derived iPSC-RGCs could help to fully understand the effects of this approach on the progression of disease pathology; however, there are challenges in finding an efficient delivery system for this application. The CRISPRa system used here would be too bulky to package in an AAV system, and it would have to be modified, potentially requiring a dual AAV strategy. Smaller Cas9 alternatives, such as Staphylococcus aureus Cas9, have been developed to circumvent this issue, allowing for the system to be packaged in a single AAV construct. These might present an alternative approach using a single vector for delivery and efficient rescue of RGCs. The development of this modified CRISPRa system for use in iPSC-RGC could help further characterise the effects of OPA1 haploinsufficiency on OPA1 isoform and l/s form ratios and test the rescue of other cellular phenotypes. Furthermore, additional analyses, such as RNA-seq, could further elucidate the downstream effect of OPA1 upregulation on patient-derived RGC survival and confirm the specificity of the regulation as part of the preclinical development of this potential therapeutic strategy.

## 4. Materials and Methods

### 4.1. CRISPR-Cas9 Gene Editing of OPA1

Wild-type HEK293T cells were gene-edited using CRISPR-Cas9 in combination with a homology directed repair template (HDR), as previously described [45]. Two hours prior to nucleofection, the cells’ media were supplemented with 10 µM of ROCK inhibitor (ROCKi; Stemcell Technologies, Vancouver, BC Canada) to aid cell survival. For nucleofection, the cells were dissociated using TrypLE (Lifetech, Grand Island, NY, USA) into a single-cell suspension, and 2 × 10^5^ cells were resuspended in Supplemented Nucleofector Solution P3 (Lonza, Basel, Switzerland). The Cas9–RNP complex was assembled by mixing the resuspended crRNA and tracrRNA in IDT Duplex Buffer (Integrated DNA Technologies) to achieve a final duplex concentration of 50 µM. This was heated at 95 °C for 5 min and allowed to cool at the benchtop for 15 min. The Cas9–RNP complex was completed with the addition of 150 pmol of 20-base-length gRNA and 125 pmol of Cas9 enzyme (Integrated DNA Technologies, Coralville, IA, USA), which was incubated for 15 min at room temperature. The final Cas9–RNP complex, 200 pmol of the HDR template (Supplementary Materials; Figure S1) and 120 pmol of Electroporation Enhancer (Integrated DNA Technologies) were added to the cell suspension, and the mixture was nucleofected using program CA-137 on a Lonza 4D-nucleofector X-unit (Lonza), Nucleofected cells were plated at varying cell densities and left to grow for two days on DMEM media with high glucose and pyruvate (Gibco, Grand Island, NY, USA) supplemented with 10% foetal bovine serum (Gibco), 0.01% Antibiotic Antimycotic (ThermoFisher, Grand Island, NY, USA), and 10 µM of ROCKi. Single-cell derived colonies were picked and plated separately, allowing for some cells to be collected for Sanger sequencing.

### 4.2. RNA Extraction, qPCR, and RT-PCR

RNA was extracted using the RNeasy Mini Kit (Qiagen, Hilden, Germany) according to manufacturer’s instructions. The RNA concentrations of the samples were quantified by measuring the absorbances at 260 nm through spectrophotometry using a Nanodrop 2000 (ThermoFisher, Grand Island, NY, USA). PCR amplification was achieved using 2X GoTaq G2 Master mix, and cDNA was synthesised using the Tetro cDNA Synthesis Kit (Bioline, Narellan, NSW, Australia; BIO-65043) by following the manufacturer’s instructions. qPCR was completed using 2X LabTAQ hi-rox green master mix (Labtech, Rotherham, UK), using *ACTIN* and *GAPDH* as reference genes. RT-PCRs were completed using cDNA obtained as previously described, and products were run on a 2% agarose gel with 10mg/mL of ethidium bromide(Eurogentec, Liège, Belgium). The uncut gel is shown in the Supplementary Figure S5. Primers are reported in the Supplementary Materials (Tables S2 and S3).

### 4.3. Western Blotting

Protein samples were extracted using radioimmunoprecipitation lysis buffer (ThermoFisher) containing 2% of Protease Inhibitor Cocktail (Sigma Aldrich, St. Louis, MO, USA). Protein quantification was performed using the Pierce Bicinchoninic acid (BCA) Protein Assay (Thermofisher) by following the manufacturer’s microplate instructions. Lysate samples were diluted with 1X NuPAGE LDS Sample Buffer (Thermofisher). The samples were run on 12% acrylamide gels. The membranes were stained with rabbit anti-OPA1 primary antibody (R&D systems, Minneapolis, MN, USA) at a dilution of 1:2000 and HRP-conjugated mouse anti-β-Actin primary antibody (Abcam, Cambridge, UK) at a dilution of 1:25,000. OPA1-stained blots were incubated with a goat-grown anti-rabbit secondary antibody (Abcam, Cambridge, UK) at a dilution of 1:8000. The membranes were imaged with Clarity Western ECL substrates (Bio-Rad, Hercules, CA, USA) for film-based membrane imaging. Uncropped Western blots are shown in Supplementary Figures S3 and S4.

### 4.4. Immunofluorescence

Cells were plated on PDL-coated glass coverslips. They were fixed with 4% formaldehyde (Thermo Sciencific, Grand Island, NY, USA), permeabilised with 0.1% Triton X-100 (Sigma, St. Louis, MO, USA), and blocked with 10% donkey serum (Sigma) and 1% BSA (ThermoFisher). The cells were incubated with rabbit anti-TOMM20 primary antibody (Santa Cruz Biotechnology) overnight at a dilution of 1:1000, followed by anti-rabbit Alexa-Fluor 647 antibody (Thermofisher) incubated together with DAPI (Sigma). The coverslips were imaged using the Leica SP8 Confocal microscope, and images were deconvoluted using HyVolution or Lightning software (version 4.7.0.28176). Single cell z-stack images were imported into ImageJ (FIJI), where the mitochondrial networks were analysed using the MiNa software, version 2.16. Networks were compared between cells plated in the same experiment to reduce variation between experiments, but the direction of change was repeatable.

### 4.5. CRISPRa Plasmid Development and Transfection

The CRISPRa machinery was encoded in a plasmid containing an inactive Cas9 fused to a VPR activator with an EGFP reporter marker purchased from VectorBuilder (Supplementary Materials, Figure S5). gRNA plasmids were designed using CHOPCHOP (https://chopchop.cbu.uib.no/, date accessed 27 January 2023) and custom ordered in VectorBuilder plasmids with U6 promoters and with an mCherry marker expressed (Supplementary Materials, Figure S6). Cells were transfected using Lipofectamine 2000 (ThermoFisher) reagent following the manufacturer’s instructions. Cells were harvested after 48 h.

## 5. Conclusions

In this study, we used gene-edited HEK293T cells to model OPA1-associated DOA and test CRISPRa. c.2708_2711del cells exhibited reduced *OPA1* mRNA transcript and OPA1 protein and increased mitochondrial network fragmentation. The data suggest that the application of CRISPRa can be used to increase OPA1 expression levels at homeostatic isoform ratios, improving mitochondrial networks. Collectively, the data suggest that CRISPRa can have a potential therapeutic benefit in OPA1-associated DOA that needs to be explored further in more physiologically relevant models, such as iPSC-RGCs or animal models.

## Data Availability

No large datasets were generated or analysed during this study.

## Supplementary Materials

The following supporting information can be downloaded at https://www.mdpi.com/article/10.3390/ijms26136364/s1.

## Abbreviations

The following abbreviations are used in this manuscript:

DOA	Dominant Optic Atrophy
RGCs	Retinal ganglion cells
CRISPR	Clustered Regularly Interspaced Short Palindromic Repeats
CRISPRa	CRISPR activation
TSS	Transcript Start Site
gRNA	guide RNA
WT	Wild-type
NMD	Nonsense mediated decay
HDR	Homology directed repair
ROCKi	ROCK inhibitor
dCas9	dead Cas9
AAV	Adeno-associated virus
ISO	Isoform
Unt.	Untransfected
bp	Base pair
TOMM20	Translocase of the outer mitochondrial membrane 20
HEK293T	Human embryonic kidney 293T cells
PAM	Protospacer adjacent motif
EGFP	Enhanced Green Fluorescent Protein
PDL	Poly-d-lysine
BSA	Bovine Serum Albumin
OXPHOS	Oxidative phosphorylation
mtDNA	Mitochondrial DNA
dSpCas9	Dead *Streptococcus pyogenes* Cas9
