# Supplementary material for: CRISPRa-Mediated Increase of OPA1 Expression in Dominant Optic Atrophy
Source: Int J Mol Sci. 2025 Jul 2;26(13):6364. doi: 10.3390/ijms26136364 (PMC12249591; doi:10.3390/ijms26136364)
Supplement: Supplementary file 1 [file ijms-26-06364-s001.zip › ijms-3688958-supplementary.pdf]

Supplementary Materials

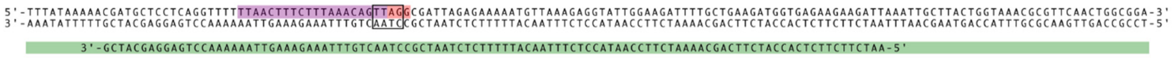

Figure S1. HDR Template highlighted in green. The PAM site is marked in red and the gRNA is in purple. The edit site is boxed in black.

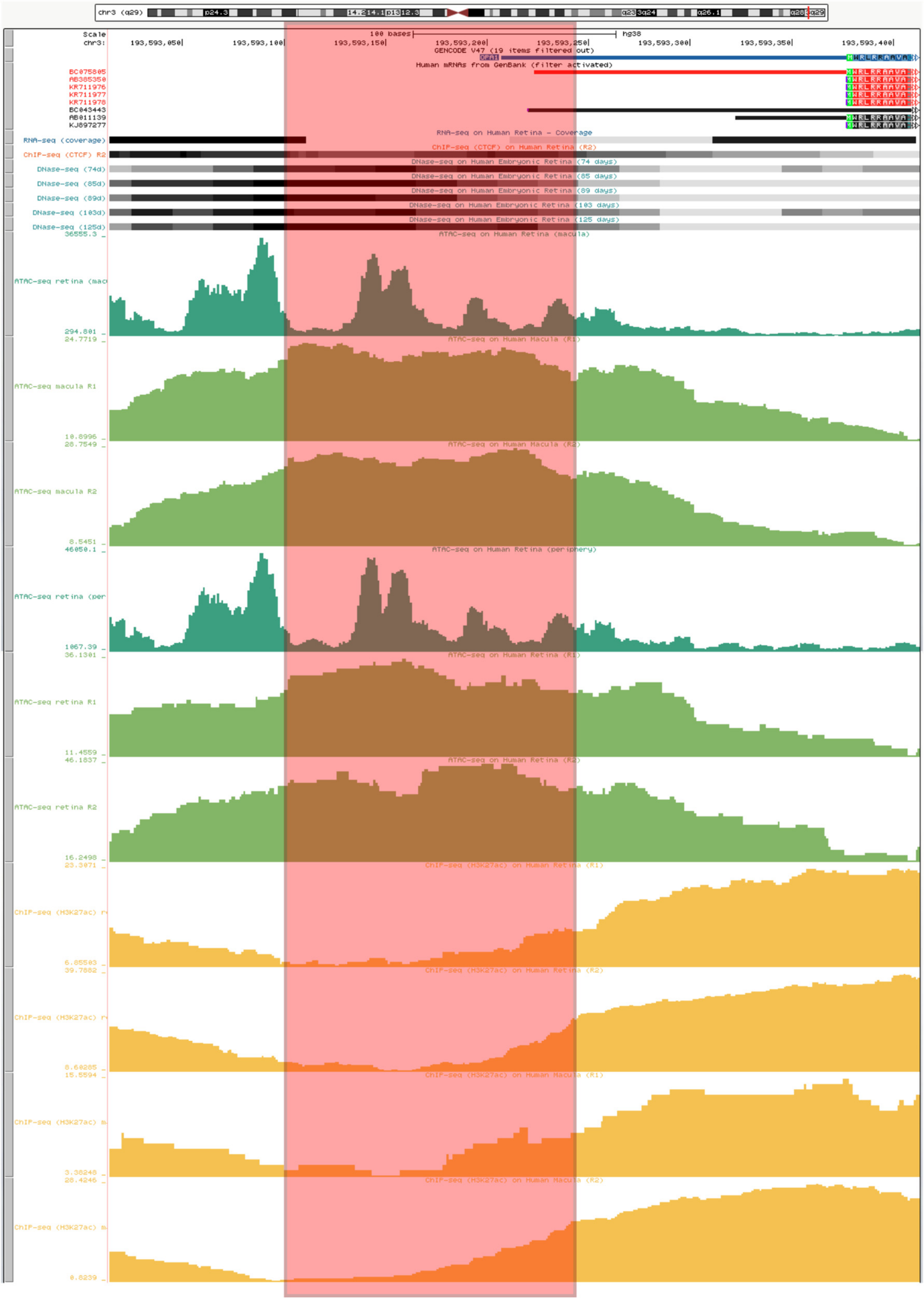

Supplementary Figure S2. Regions of transposable-accessible chromatin (ATAC-seq) in adult human retina. The data was obtained from the multi-omics database RegRet, mapped on human genome build hg38 (accessed 30/01/2025). gRNAs were designed in the region marked in red

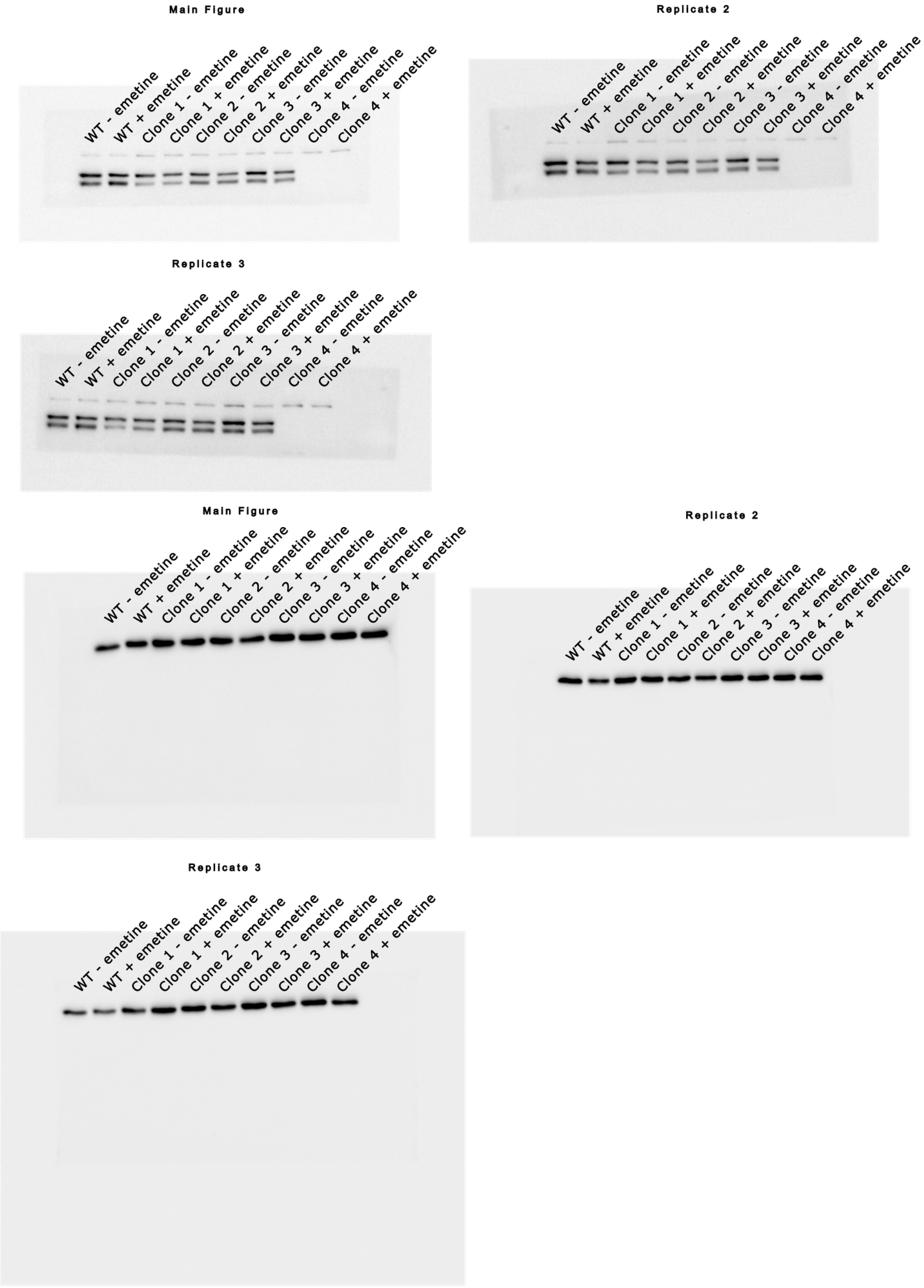

Supplementary Figure S3. Uncropped Western blots for Figure 1. Upper three blots were stained with anti-OPA1 (R&D systems, Minneapolis, MN, USA) while the lower three blots were stained with anti-β-Actin (Abcam, Cambridge, UK).

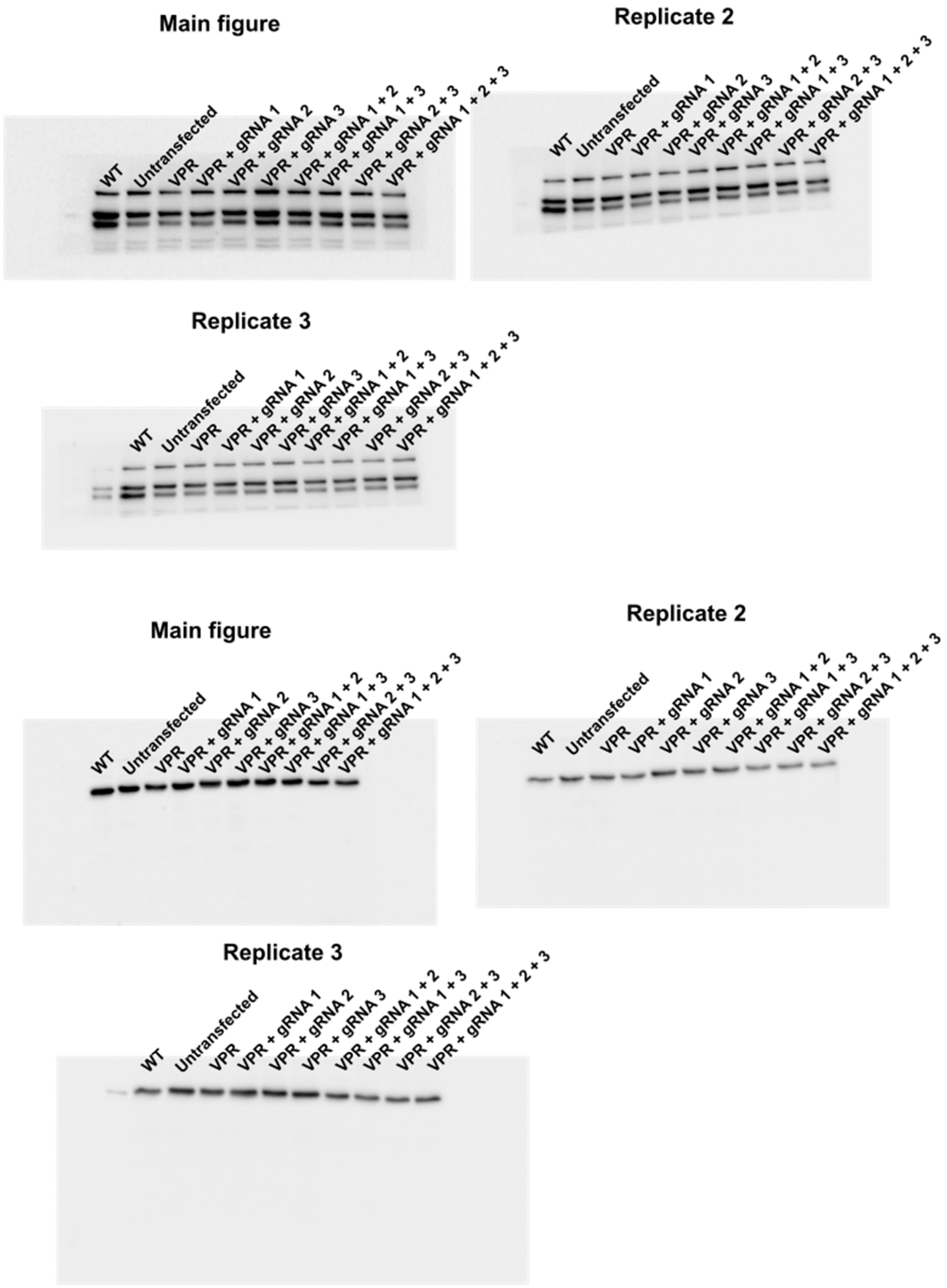

Supplementary Figure S4. Uncropped Western blots for Figure 3. Upper three blots were stained with anti-OPA1 (R&D systems, Minneapolis, MN, USA) while the lower three blots were stained with anti-β-Actin (Abcam, Cambridge, UK).

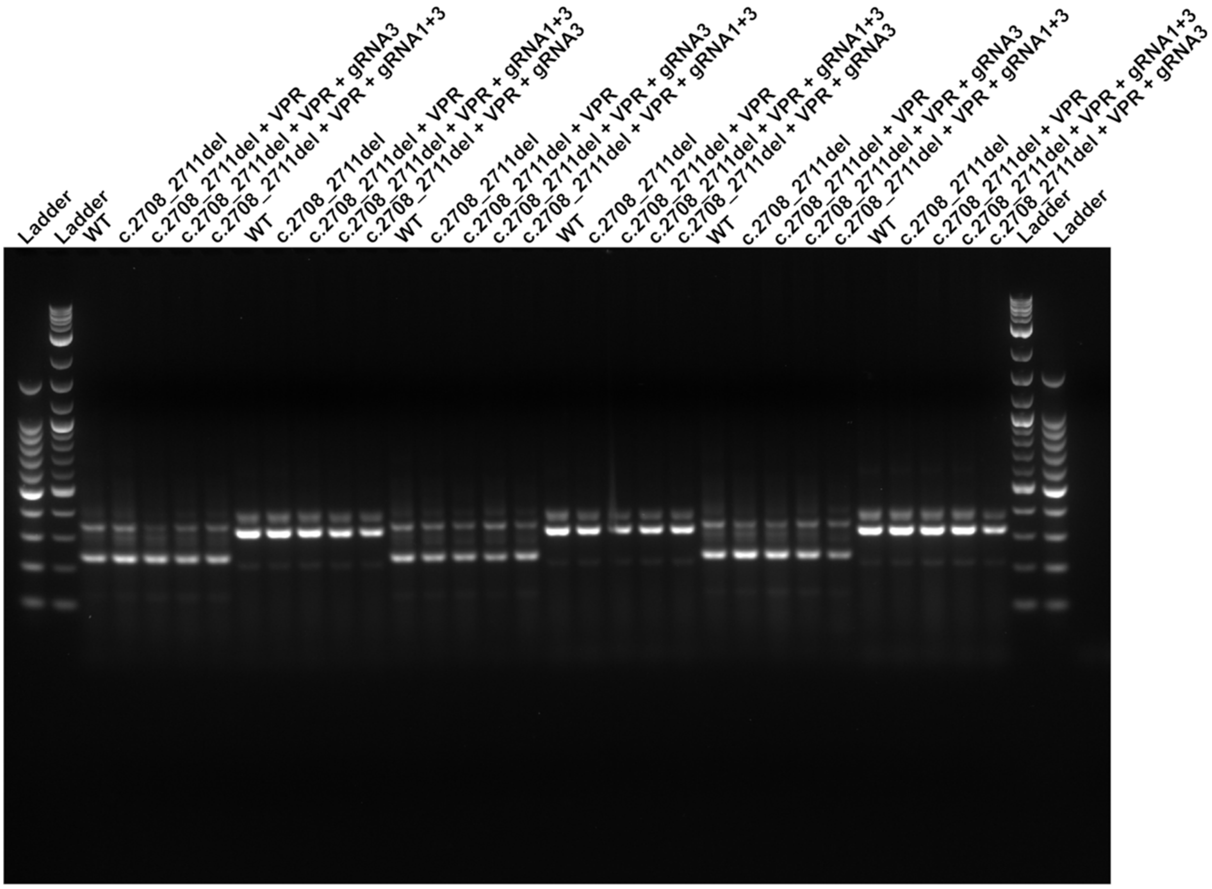

Supplementary Figure S5. Uncropped Ethidium bromide stained gel with RT-PCR samples shown in Figure 4. The gel shows the 3 biological replicates

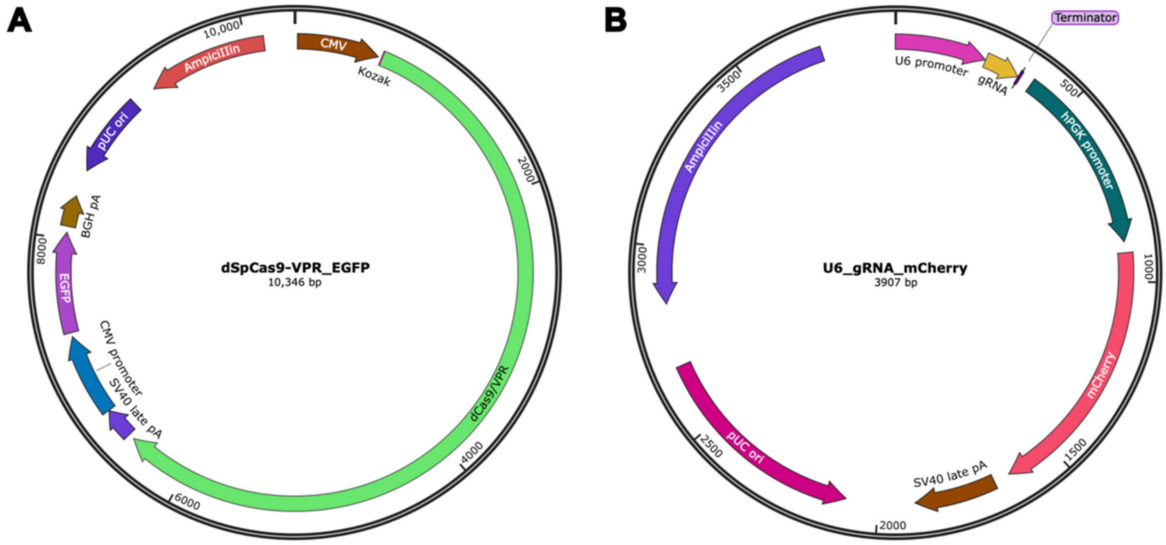

Figure S6. CRISPRa plasmids. A. Plasmid with an inactive Cas9, fused to a VPR activator, under a CMV promoter with an EGFP reporter marker. **B.** Plasmid containing a gRNA under a U6 promoter, with an mCherry reporter marker.

Table S1. 2way ANOVA results comparing (-) and (+) emetine treated samples from Figure 1.

Šídák's multiple comparison test	Predicted (LS) mean diff.	Adjusted P Value
WT	-0.07559	0.9936
Clone 1	-0.1833	0.7748
Clone 2	-0.3421	0.1962
Clone 3	-0.1624	0.8485
Clone 4	-0.3429	0.3801

Table S2. qPCR primers

Gene	Primers		
	Forward	Reverse	Size (bp)
OPA1	CGACCCCAATTAAGGACAT	GCGAGGCTGGTAGCCATAT	102
ACTIN	CCAACCGCGAGAAGATGA	CCAGAGGCGTACAGGGATAG	97
GAPDH	CCCCACCACACTGAATCTCC	GGTACTTTATTGATGGTACATGAGA	107

Table S3. RT-PCR primers

	Primers	
	Forward	Reverse
**Δ5b**	TGCCTGACATTGTGTGGGAA	TGCCTTTGTCATCTTTCTGCAA
**With 5b**	TGCCTGACATTGTGTGGGAA	TCTGTTGGGCATAGCTCGTG
